# Post-infection viral superinfection technology could treat HBV and HCV patients with unmet needs

**DOI:** 10.1186/s41124-017-0028-x

**Published:** 2018-01-05

**Authors:** Tibor Bakacs, Rifaat Safadi, Imre Kovesdi

**Affiliations:** 1 HepC, Inc., Budapest, Hungary; 2 Hadassah Hebrew University Medical Center, Jerusalem, Israel; 3 ImiGene, Inc., Rockville, MD USA

**Keywords:** Chronic HBV/ HCV, DAA agents, Viral superinfection, dsRNA virus, Antiviral gene responses, Pandemic preparedness

## Abstract

**Background:**

Viral hepatitis deaths from acute infection, cirrhosis, and liver cancer have risen from the tenth to the seventh leading cause of death worldwide between 1990 and 2013. Even in the oral direct acting antiviral (DAA) agent era there are still large numbers of patients with unmet needs. Medications approved for treatment of chronic hepatitis B virus (HBV) infection do not eradicate HBV often requiring treatment for life associated with risks of adverse reactions, drug resistance, nonadherence, and increased cost. Although DAAs increased virologic cure rates well over 90% in all hepatitis C virus (HCV) genotypes, HCV infection still cannot be cured in a small but significant minority of patients. While most of the medical issues of HCV treatment have been solved, the current costs of DAAs are prohibitive.

**Results:**

The post-infection viral superinfection treatment (SIT) platform technology has been clinically proven to be safe and effective to resolve acute and persistent viral infections in 42 HBV and HCV patients (20 HBV, 22 HCV), and in 4 decompensated patients (2 HBV, 2 HCV). SIT employs a non-pathogenic avian double stranded RNA (dsRNA) virus, a potent activator of antiviral gene responses. Unexpectedly, SIT is active against unrelated DNA (HBV) and RNA (HCV) viruses. SIT does not require lifelong therapy, which is a major advantage considering present HBV treatments. The new viral drug candidate (R903/78) is homogeneously produced by reverse genetics in Vero cells. R903/78 has exceptional pH and temperature stability and also excellent long-term stability; therefore, it can be orally administered, stored and shipped without freezing. Since R903/78 is easy to stockpile, the post-infection SIT could also alleviate the logistic hurdles of surge capacity in vaccine production during viral pandemics.

**Conclusion:**

To help large number of HBV and HCV patients with unmet needs, broad-spectrum antiviral drugs effective against whole classes of viruses are urgently needed. The innovative SIT technological platform will be a great additional armament to conquer viral hepatitis, which is still a major cause of death and disability worldwide.

## Background

Viral hepatitis deaths from acute infection, cirrhosis, and liver cancer have risen from the tenth to the seventh leading cause of death worldwide between 1990 and 2013, HBV and HCV accounting for 96% of viral hepatitis-related mortality. Although biomedical advances have led to efficacious vaccines and treatments for HBV and HCV that could be delivered at scale, mechanisms to fund these interventions in the poorest countries are largely non-existent. The small proportion of global health funding targeted at viral hepatitis is disproportionate to its importance as a major cause of death and disability [1].

Approximately 5% of the world population is chronically infected with HBV and close to 700 thousand people die every year due to complications of hepatitis B, including cirrhosis and liver cancer [2]. A recent meta-analysis of 59 studies on antiviral therapy for chronic HBV infection in adults reported that medications approved for treatment of chronic HBV infection do not eradicate HBV [3]. Therefore, treatments should be administered for many years and often for life, which are associated with risks of adverse reactions, drug resistance, nonadherence, and increased cost. Importantly, despite the availability of a few therapeutic options, patients most in need with decompensated HBV liver cirrhosis suffer from various severe, often life threatening complications including portal hypertension, gastrointestinal variceal bleeding, ascites and hepatic encephalopathy. About a quarter of such patients die within 1 year [4]. Although a safe and effective prophylactic HBV vaccine for infants had already been introduced nationwide in 185 countries (thus, global coverage of HBV vaccine is ~83%),^1^ due to the 350–400 million chronically infected people hepatitis B will remain a tenacious scourge for the foreseeable future that require better treatment options.

Curing HCV infection has become a reality with current DAA drugs. The second generation of DAAs including sofosbuvir (Sovaldi), simeprevir (Olysio), and fixed combination medicines Harvoni and Viekira Pak increased cure rates to over 90% without the need for interferon and effectively treat all HCV genotypes [5]. Notwithstanding, HCV infection still cannot be cured in a small, but significant minority of patients. There are several reasons for this. HCV resistance to DAAs has for example an important role in the failure of interferon-free treatment regimens [6]. Furthermore, the progression of esophageal varices seems to be independent of the virologic response to therapy. Although PEG-IFN-free DAA combo regimens will eventually increase the rate of virologic cure to nearly 100%, it remains to be assessed whether this will be translated into a universal clinical benefit. Therefore, the blanket assumption that eliminating HCV at all stages will ultimately result in removing the disease burden of HCV cirrhosis is, as yet, an unproven clinical and pharmacoeconomic extrapolation [7]. In addition, DAA therapy is associated with high mortality in patients with Child’s C cirrhosis. It is likely that some patients with decompensated cirrhosis had reached the “point of no return”, where DAA therapy is less effective in improving liver function [8, 9]. Importantly, HCV-associated disease burden will remain substantial even in the oral DAA era, as a recent US study predicted [10]. When 1.8 million HCV patients will have received DAA treatment from the launch of oral DAAs in 2014 until 2030, 320,000 patients will still die, 157,000 will develop hepatocellular carcinoma, and 203,000 will develop decompensated cirrhosis in the next 35 years.

A recent cause of great concern is that DAA treatment could increase the risk of hepatocellular carcinoma in cirrhotic HCV patients [11, 12]. Consistent with this, a study based on real-world clinical practice suggested that rapid viral suppression by IFN-free DAAs may depress cytotoxic inflammatory cells, thus leading to a state of temporal relative immunosuppression, not only to transformed cells, but also to cells co-infected with viruses [13–15]. These facts justify alternative antiviral management, such as our superinfection technology that stimulates the native immune system of the host, particularly in advanced cirrhosis cases who are at high risk for HCC.

While most of the medical issues of HCV treatment have been solved, the current costs of DAAs are prohibitive. With an estimated 130–150 million people worldwide infected with HCV, the global treatment gap is reminiscent of the early AIDS crisis, since most countries lack access to curative medicines. The goal to eliminate hepatitis as a major public health threat by 2030 is therefore only achievable through planning urgent and affordable access to essential medicines—in all countries [16]. To this end, generic drugs are developed, the cost of which will soon be very affordable even for developing countries. For example, a HCV treatment that costs less than $300 – a tiny fraction of the $80,000-plus price charged by major drugmakers – has been successfully tested in Egypt (the worst affected country in the world, where 10% to 15% of the population has hepatitis C). It is expected to be available within two years. Three-quarters of people with hepatitis C, who live in middle-income countries, stands to benefit from such efforts.^2^

The development of affordable viral superinfection therapy (SIT), which is a revolutionary new platform technology approach using an entirely different modality from the DAA drugs currently in pipelines, could complement the generic DAA drug development efforts. SIT could be developed into a safe, effective and affordable medicine for both HBV and HCV patients with unmet needs as described below.

### Beyond DAAs

#### The idea of viral superinfection therapy

Viral superinfection therapy exploits viral competition for the treatment of acute and persistent viral infections. The idea is based on the clinical observation that unrelated viruses might interact in co-infected patients. Hepatitis infection by one type of virus (e.g. HCV) is often abolished following accidental infection by a second hepatitis virus (e.g. HBV). The dominant virus interferes with the replication of the other virus. Nevertheless, in cases when both viruses are pathogenic the disease persists and hepatitis remains. However, the patient may benefit from superinfection with a non-pathogenic dsRNA virus such as the infectious bursal disease virus (IBDV), which is a potent activator of the interferon-dependent antiviral gene program. Because of its major economic importance to the world’s poultry industries, attenuated IBDV strains are used as commercial vaccines, and some of them are propagated in Vero cells. These vaccines have excellent safety record [17]. While wild type IBDV is a highly contagious disease of young chickens characterized by immunosuppression and mortality generally at 3 to 6 weeks of age, the attenuated vaccine strains cause no disease. Furthermore, even the wild type IBDV is not known to be a hazard in transmitting to other species despite its worldwide distribution in the domestic fowl [18, 19]. Therefore, viral competition was exploited using a non-pathogenic, attenuated vaccine strain of IBDV to resolve acute and persistent HBV or HCV infections. In the above context, we also discussed in the past an intentional superinfection strategy for the control and treatment of AIDS in view of the improved survival of HIV infected patients naturally infected with the GB virus C [20, 21].

#### The proof-of-concept of viral superinfection in animals and patients

The proof of SIT concept was first demonstrated in marmoset monkeys. Animals were infected with human hepatitis A virus and then 1 and 3 weeks later they were superinfected with an attenuated IBDV. The superinfected monkeys did not show the characteristic serum glutamic pyruvic transaminase (SGPT) elevation and their liver biopsies showed no pathologic changes, while the control animals exhibited six times higher SGPT enzyme levels than the superinfected groups and hepatitis was detected by histopathology. This experiment proved for the first time that using of an apathogenic virus for the cure of a virus-induced disease is a realistic possibility [22].

Then, the proof of SIT concept was demonstrated in a preliminary clinical trial that included 84 patients of both sexes (14–70 years), with either a diagnosis of acute B (43 patients) or acute C (41 patients) viral hepatitis [23]. Patients were hospitalized because of jaundice, other clinical signs of acute hepatitis (fever, severe malaise, loss of appetite), and a 10 to 100-fold elevation of alanine-aminotransferase (ALT) level. The diagnosis of HBV infection was verified by the presence of HBsAg, HBeAg, and anti-HBcIgM antibody. Acute HCV infection was determined by the exclusion of A and B virus, EBV, and CMV infection and by the appearance of anti-HCV antibody. The patients received an intranasal therapeutic vaccine (V903/78 strain) containing live attenuated IBDV once every day (4000 IU/day; 4 × 10^6^ TCID_50_)^3^ for a week, then 3 times a week for two weeks, and finally once a month for 6 months. Criteria of remission was the normalization of serum bilirubin and ALT levels, disappearance of HBsAg, and no relapse within 6 months.

Significant difference was observed between the IBDV treated and control groups as only 9% of the HCV and none of the HBV patients progressed into chronic disease, whereas 13% and 26% of the controls, respectively, did (Table 1). The percentage of HCV CAH control patients (26%) is lower than expected (75%). This discrepancy might be, at least partly, explained by the fact that the trial HCV patients do not represent the general HCV population, as these patients were hospitalized due to jaundice and clinical symptoms (thus, comprising only 20% to 30% of adults with acute HCV infection). It is known that the rate of chronic HCV infection is lower in patients who develop jaundice or symptoms during the acute onset of HCV infection as compared to those who are anicteric [24]. Furthermore, considering that several of the relapsed HCV patients eventually also progressed into CAH (unpublished observation), the discrepancy between the trial results and real life becomes smaller. Prior to complete recovery 9% of HBV and 79% of HCV control patients, but only 5% and 32% of the IBDV treated patients relapsed. Late remissions (requiring more than 6 months) were recorded significantly more frequently in both HBV and HCV control groups (17% and 42% respectively in controls but 0% and only 14% with IBDV treatment). While remission within one month of treatment was registered more often in the virus treated groups (both 50%), than in the control groups (26% and 21%, HBV and HCV, respectively). The duration of the first icteric phase was also shortened with the IBDV treatment (by 20% in the HBV and 40% in the HCV groups). No serious adverse events related to superinfection treatment were recorded.

**Table 1:** Response rates of an IBDV therapeutic vaccine on acute HBV and HCV infections

Response	HBV		HCV	
	IBDV	control	IBDV	control
Progression into CAH^b^	0/20 (0%)	3/23*^a^ (13%)	2/22 (9%)	5/19* (26%)
Relapses	1/20 (5%)	2/23 (9%)	7/22 (32%)	15/19*** (79%)
Late remission^c^	0/20 (0%)	4/23** (17%)	3/22 (14%)	8/19** (42%)
Fast remission^d^	10/20 (50%)	6/23 (26%)	11/22 (50%)	4/19 (21%)
Duration^e^ (weeks ± SD)	5.9 ± 3.0	7.5 ± 3.7	5.3 ± 4.4	8.9 ± 7.4

^a^Significance (*p* value, chi-square, Yates’ correction): *p < 0.05; **p < 0.02; ***p < 0.01;

^b^Chronic active hepatitis

^c^Remission over 6 months

^d^Remission within 1 month

^e^Duration of the first icteric phase

**Fig. 1:**
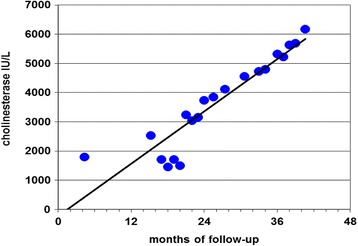
Cholinesterase activity levels indicating liver regeneration in a chronic HCV patient treated with IBDV; Legend: A striking feature of the superinfection therapy (SIT) was the regeneration of the cirrhotic liver over several years of follow up

Most importantly, SIT was also safe and effective in four parenchymally decompensated chronic hepatitis patients (two with HBV, and two with HCV), with various life-threatening complications, e.g. portal hypertension, diuretic-resistant ascites, progressive jaundice, generalized edema, hepatic encephalopathy, etc. All four patients went into long-lasting remission or were stabilized with spectacular clinical improvement with the IBDV treatment, while conventional therapy failed to stabilize the patients’ conditions. No treatment associated toxicity was reported. A striking feature of SIT was the regeneration of the cirrhotic liver over several years of follow up (Fig.1) [20, 25, 26].^4^ ^5^

Despite the success of the clinical trials, development of SIT was abandoned in the 90s due to an unresolved regulatory issue hindering approval. Specifically, reproducible manufacture of homogeneous IBDV drug substance that satisfies both FDA and EMA regulatory requirements could not be met by conventional virus production.

### Re-establishment of superinfection therapy by reverse genetics

#### Reproducible manufacture of homogeneous new biologic drug candidate

Procedures developed during the 1990s to genetically manipulate the genomes of negative-strand RNA viruses and to rescue infectious viruses entirely from cloned cDNAs, commonly referred to as reverse genetics, have revolutionized the analyses of viral gene expression, viral replication and pathogenesis. They have also paved the road for engineering of these viruses for vaccine and gene therapy development [27].

**Fig. 2:**
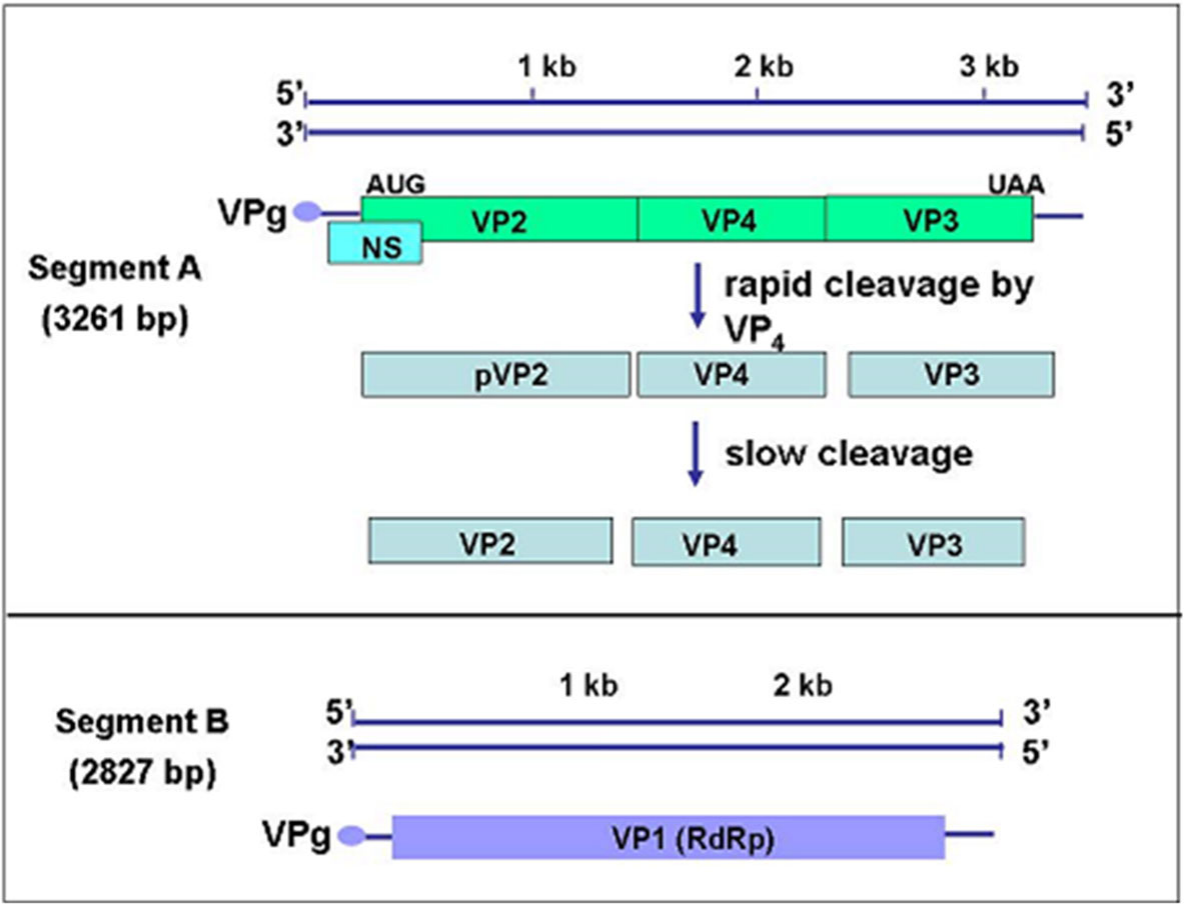
Genome organization and proteins of infectious bursal disease virus; *Legend*: Segmented dsRNA virus, such as IBDV, can be recovered from its cloned cDNAs of genomic segments A and B

Use of IBDV as an agent against a human disease requires a well characterized drug candidate. Therefore, we have cloned the V903/78 vaccine strain^6^ and assembled it into cDNA plasmids allowing reproducible viral production. Phylogenic relations to other IBDV strains placed this virus within the tissue adapted vaccine strains with closest relationship to D78. It has been also shown that segmented dsRNA virus, such as IBDV, can be recovered from its cloned cDNAs of genomic segments A and B (Fig.2) [28–30].

In order to remove the single most important regulatory hurdle a new biologic drug candidate, R903/78, was created by using reverse genetics. Full-length cDNA clones of IBDV segments A and B of strain V903/78 were constructed using strain D78 as a template. Nucleotide changes were incorporated into the correct PCR fragments generating the R903/78 cDNA plasmids. Both plasmids were sequenced to confirm the identity of segment A and segment B. Viruses were recovered from transfected Vero cells [31].

#### Physical and chemical properties of R903/78

R903/78 was manufactured using green monkey kidney Vero cells by contract manufacturing organization (CMO) (Vibalogics GmbH) and the final product was formulated in 10% buffered sucrose. IBDV production is very simple as the virus is secreted into the media. Oral use only requires minimum level of purification consisting of filtration, concentration and buffer exchange. Assays for potency (infectious titer, virus particle enumeration, biological activity) and safety (sterility, mycoplasma, bioburden, endotoxin, virus contamination, in vivo adventitious agents, general safety, particulates) as well as additional tests to assess purity were developed.

Several human cell lines supported IBDV propagation in the absence of visible cytopathic effect. The virus was stable from pH 6 to pH 8 and demonstrated significant resistance to low pH and also proved to be highly resistant to high temperatures. R903/78 can be stored at or below +5°C for at least 6 months and at room temperature (+22 °C) at least 4 h. R903/78 has demonstrated remarkable stability under a variety of conditions during controlled stability studies [31]. Virus stability indicates that liquid formulation would be suitable for distribution in developed countries, but for developing countries a freeze-dried product would be more convenient.

R903/78 was not toxic in rodents in doses 400 times that of proposed for human trials. Single and multiple oral administration of IBDV elicited antibodies with neutralizing activities in vitro. However, repeat oral administration of R903/78 was successful despite the presence of neutralizing antibodies. Single oral and intravenous administration indicated that IBDV does not replicate in mammalian liver alleviating some safety related concerns. These data supports the development of an orally delivered anti-HBV and anti-HCV virus based drug agent for human use [31].

#### Analysis of virus-activated, interferon related gene expression changes after IBDV treatment

The innate immune system senses viral nucleic acid invading mammalian cells and triggers type I interferon production. Genes that are part of the pathway regulators efficiently modulate the innate immune response to counteract viral infection. IBDV is a double stranded RNA virus and expected to induce a very strong interferon (INF) response. To this end, we monitored the expression changes of 17 virus-inducible genes associated with INF response in the liver of mice following R903/78 treatment. At 0 min mice were treated with 1 million IBDV particles intravenously via the tail vein, and then they were sacrificed at 2 h, 4 h, 8 h, 16 h, 24 h, 72 h, 1 week, and liver RNA was determined by real time quantitative PCR. Highest over expression was found in IRF7 (up to 267 x baseline), ZBP1 (up to 98 x baseline), TLR9 (up to 12 x baseline), Ifi204 (up to 22 x baseline) and TLR3 genes (up to 13 x baseline) within 4 to 8 h after infection.

#### The safety aspects of the R903/78 drug candidate

More distantly donor and recipient host species are related, the more difficult it is for any virus to jump between those species and establish a productive infection. Key components of the virus–host interaction in birds and mammals diverged along with their hosts during more than 200 million years such that 13 mutations may be required for avian influenza viruses to establish productive infections in humans [32, 33]. For such gigantic jump influenza virus requires an intermediate host, the swine, to pre-adapt to humans. Other avian viruses, e.g. NDV or IBDV, do not have such natural hosts. Not unexpectedly, no zoonosis cases were ever reported in workers of chicken coops and/or IBDV vaccine production facilities over the past 50 years during IBDV mass vaccination programs in poultry. Consistent with this, repeated oral administration of large IBDV doses (up to a cumulative dose of 3 × 10^9^ infective particles) was required to maintain artificial viremia and achieve long-lasting remission in several advanced chronic decompensated hepatitis patients. Notwithstanding, even a very low risk of zoonosis is a legitimate concern for the regulatory authorities. Therefore, reverse genetics technology is used in order to produce batch to batch consistency without the need to plaque-purify the IBDV drug candidate and prevent spontaneous mutations.

We emphasize that no serious side effects were observed during IBDV superinfection therapy even in parenchymally decompensated moribund patients. This is in stark contrast to systemic IFN-based therapy, which is associated with a wide array of adverse effects. Neuropsychiatric side effects such as depression and irritability are the most troublesome that may require dose modification or even discontinuation of therapy [34]. One possible explanation could be the very different target range of these two therapies. Receptors for the type I and II IFNs are found on the surface of most cell types such that systemic IFN therapy has an almost ubiquitous nature of signaling [35]. While one of the outstanding characteristics of viruses is their very restricted cellular and host tropism [36]. A further major difference between systemic IFN-based and superinfection therapy is that following interaction of IBDV with appropriate cells, its dsRNA is recognized by specific receptors (e.g. TLR3), which activate *several* gene families from within. The number and types of genes that are modulated by IBDV superinfection will be evaluated more precisely in future studies, but it is already clear that the two therapeutic modalities are not the same.

Concerning the effect of IBDV superinfection on integrated HBV DNA into the infected host’s hepatocyte genome, we can only speculate as both of our HBV infected decompensated patients reacted positively to the SIT therapy, but their HBV integration status was not evaluated. It has been demonstrated that integration of HBV has the primary *cis* effect of altering gene regulation [37]. Sequence variations and structural alterations of the HBV genome generate novel HBx-human chimeric proteins that may exert a *trans* effect by facilitating host immune surveillance evasion and/or contribute to tumorigenesis. We hypothesize that induction of several innate immune system gene families by the dsRNA of IBDV is capable counteracting immune surveillance evasion more effectively than systemic IFN therapy can do. Future clinical studies need to evaluate HBV integration status and correlate with the efficacy of treatment.

#### The estimated cost of viral superinfection therapy

At this point in time it is difficult to estimate the exact cost of SIT. Although drug prices have very little to do with manufacturing costs, manufacture of IBDV is probably one of the simplest and cost effective for a biological drug requiring only filtration technology. Regulatory requirements are further simplified as it is an oral biological. On the negative side SIT will require individual dosing regiments which necessitate the definition of good clinical endpoints. However, costs can be further substantially reduced as the same drug can be used against several acute and chronic virus infections including important pandemic targets. Also the development of a lyophilized formulation would allow possible widespread use in developing countries.

## Conclusions

Rare cancer successes spawned ‘exceptional’ research efforts, as in many clinical trials that failed to help enough patients, there were exceptions, rare patients with advanced cancer whose tumors shrank or even disappeared for many months or years [38]. NCI chief Harold Varmus stated that we can really learn from such “outlier” cases, “exceptional responders” since they may explain why a drug sometimes has dramatic beneficial effects in certain patients, which in turn could allow more people to benefit from it. In our view, the published cases of the 4 parenchymally decompensated moribund patients with HBV and HCV infections should also instigate further research efforts to allow benefiting many millions of hepatitis patients worldwide with unmet needs.

Clearly, the currently used “one bug, one drug” treatment approach (e.g. DAA drugs) is inadequate to tackle the unmet needs of these hepatitis patients. Broad-spectrum antiviral drugs effective against whole classes of viruses are urgently needed. As interferon is active against most vertebrate-infecting viruses, SIT could be developed into the first technological platform, which will be registered for the “one drug, multiple bugs” treatment approach of viral diseases *Science* called for [39].

There are more than 17,500 hepatitis patients on the liver transplantation waiting list just in the USA, with more added each day. Almost 5000 patients receive transplanted livers every year, but more than 1700 patients die each year while on the waiting list.^7^ The superinfection therapy, which was proved to be safe and effective in parenchymally decompensated HBV and HCV patients, respectively, may be able to save the life of patients on the waiting list. In addition, SIT could also give hope to those patients, who will be diagnosed too late to benefit from DAA therapy because with decompensated cirrhosis they had reached the “point of no return”, where DAA therapy is less effective in improving liver function.

Since the R903/78 viral agent is easy to produce, store and stockpile, SIT could also be developed into a general post-infection viral therapy. This could become a plan “B” alleviating the logistic hurdles of surge capacity in vaccine production and increasing international pandemic preparedness [40, 41]. These predictions could be confirmed or refuted in controlled clinical trials recruiting HBV and HCV hepatitis patients with unmet needs. However, advancing the regulatory path on hepatitis B virus treatment and exposing patients to potentially risky new interventions for a disease for which safe and effective (even if lifelong) treatment is available, requires careful consultation with stakeholders and ethical review [42]. Hopefully, our arguments for cautious superinfection clinical trial will be considered by the medical community in order to reach unequivocal conclusions about the utility of this innovative modality, particularly in HBV infections with a possibility of viral eradication during a finite treatment course.

## Abbreviations

DAA: Direct acting antiviral agent
dsRNA: Double stranded RNA
HBV: Hepatitis B virus
HCV: Hepatitis C virus
SGPT: Serum glutamic pyruvic transaminase
SIT: Superinfection treatment
SVR: Sustained virological response

## Footnotes

1
http://www.who.int/mediacentre/factsheets/fs378/en/

2
https://www.theguardian.com/society/2016/apr/13/hepatitis-c-treatment-drugs-under-300-dollars-coming-soon

3 TCID = tissue culture infective dose

4 As of April 2015, two patients (1 HBV and 1 HCV) were still well (personal communication). One HCV patient was well and working until his death in 2013; whereas one HBV patient died of liver cancer in 2003 five years following SIT had been completed.

5 It is important to note that the published decompensated HBV patient, who is well and working without any HBV medications, had a very poor prognosis before SIT was started as abdominal ultra-sound demonstrated portal hypertension. Since there was no hope that she would be able to return to work she received disabled status. She was successfully treated with IBDV (see *Case 2* in 25. Csatary LK, Schnabel R, Bakacs T. Successful treatment of decompensated chronic viral hepatitis by bursal disease virus vaccine. Anticancer Res. 1999;19(1B):629–33.). During her last check up (in 2015) she had normal liver enzyme values and her liver ultrasound showed normal liver anatomy.

6 GenBank files: JQ411012.1 and JQ411013.1

7
https://www.ucsfhealth.org/treatments/living_liver_donor_transplant/
